# Multiple causes of death analysis of chronic diseases: the example of diabetes

**DOI:** 10.1186/s12963-015-0056-y

**Published:** 2015-08-25

**Authors:** Ugo Fedeli, Giacomo Zoppini, Carlo Alberto Goldoni, Francesco Avossa, Giuseppe Mastrangelo, Mario Saugo

**Affiliations:** Epidemiological Department, Veneto Region, Passaggio Gaudenzio 1, Padova (PD), 35131 Italy; Section of Endocrinology, Diabetes and Metabolism, Department of Medicine, University of Verona, Piazzale Stefani 1, Verona (VR), 37126 Italy; Department of Public Health, Local Health Unit, Modena, Strada Martiniana 21, Baggiovara, Modena (MO), 41126 Italy; Department of Cardiac, Thoracic and Vascular Sciences, University of Padova, Via Giustiniani 2, Padova (PD), 35128 Italy

## Abstract

**Background:**

Identifying a single disease as the underlying cause of death (UCOD) is an oversimplification of the clinical-pathological process leading to death. The multiple causes of death (MCOD) approach examines any mention of a disease in death certificates. Taking diabetes as an example, the study investigates: patterns of death certification, differences in mortality figures based on the UCOD and on MCOD, factors associated to the mention of diabetes in death certificates, and potential of MCOD in the analysis of the association between chronic diseases.

**Methods:**

The whole mortality archive of the Veneto Region-Italy was extracted from 2008 to 2010. Mortality rates and proportional mortality were computed for diabetes as the UCOD and as MCOD. The position of the death certificate where diabetes was mentioned was analyzed. Conditional logistic regression was applied with chronic liver diseases (CLD) as the outcome and diabetes as the exposure variable. A subset of 19,605 death certificates of known diabetic patients (identified from the archive of exemptions from medical charges) was analyzed, with mention of diabetes as the outcome and characteristics of subjects as well as other diseases reported in the certificate as predictors.

**Results:**

In the whole mortality archive, diabetes was mentioned in 12.3 % of death certificates, and selected as the UCOD in 2.9 %. The death rate for diabetes as the UCOD was 26.8 × 10^5^ against 112.6 × 10^5^ for MCOD; the UCOD/MCOD ratio was higher in males. The major inconsistencies of certification were entering multiple diseases per line and reporting diabetes as a consequence of circulatory diseases. At logistic regression the mention of diabetes was associated with the mention of CLD (mainly non-alcohol non-viral CLD). In the subset of known diabetic subjects, diabetes was reported in 52.1 %, and selected as the UCOD in 13.4 %. The probability of reporting diabetes was higher with coexisting circulatory diseases and renal failure and with long duration of diabetes, whereas it was lower in the presence of a neoplasm.

**Conclusions:**

The use of MCOD makes the analysis of mortality data more complex, but conveys more information than usual UCOD analyses.

## Introduction

Cause-specific mortality data are of paramount importance to describe the health profile of a population, to set priorities for health policy makers, and can be used to evaluate the impact of preventive interventions. Moreover, cause-specific mortality represents a commonly adopted end-point for epidemiologic studies and clinical trials, and constitutes an essential information source to build disease registries.

Usually, reported mortality data are limited to a single cause of death, the underlying cause of death (UCOD), which is selected from all diseases mentioned in the death certificate according to international coding rules. An additional approach is represented by the analysis of all conditions reported in the certificate (multiple causes of death – MCOD), to assess any mention of a disease irrespective of its selection as the UCOD.

The adoption of MCOD analyses is necessary to capture the multiple diseases that can lead to chronic disease mortality, such as diabetes-related mortality [1, 2]. Diabetes is associated with several conditions resulting in an increased risk of death, mainly cardiovascular disorders, renal failure, and infectious diseases; furthermore, recently the association has emerged between diabetes and mortality from various forms of cancer [3, 4], and from chronic liver disease (CLD) [5].

Important issues associated to certification practices arise when dealing with diabetes-related mortality. The first is the mention of diabetes on the death certificate: although diabetes might not be reported because it really did not contribute to death, previous studies found that patients’ demographics, place of death, role of the certifying physician, duration and treatment of diabetes, and associated diseases influenced the probability of mention of diabetes [6–11]. However, such studies were limited to the US and UK, and were mainly carried out on small samples of known diabetic subjects. Another problem is placement of diabetes within the death certificates: it heavily influences the probability of its selection as the UCOD, and has been demonstrated to largely vary by country [12]. MCOD data allow problems to be identified in the process of recording and elaborating information on death certificates [13], and are fundamental to elucidating heterogeneity between countries in the selection of chronic diseases, including diabetes, as the UCOD [14, 15]. Indeed, mortality rates from diabetes, judged only from the UCOD, can fluctuate over time due to trends in certification practices; since MCOD provide the most consistent data on time trends [16], both MCOD and UCOD mortality rates should be examined to properly interpret changes in cause-specific mortality [17, 18].

Lastly, MCOD can be analyzed to explore associations between different diseases leading to death; however many intricacies arise when associations found between conditions mentioned in the certificate are assumed as causal relationships at the population level [19].

The present study explores the limits and potential of MCOD analysis in the assessment of the mortality burden from diabetes in northeastern Italy, with the four following specific aims:

1. to examine patterns of the mention of diabetes in different sections of the death certificate, and the associated probability of selection of diabetes as the UCOD;
2. to compute mortality rates and proportional mortality for diabetes, comparing figures based on the UCOD and on MCOD;
3. to measure the rate and test factors associated to the mention of diabetes in a subset of death certificates of decedents from a cohort of known diabetic subjects;
4. to assess the potential of MCOD data in the analysis of associations between different diseases reported in the certificate, even when the knowledge about such relationship is still limited within the medical community. As an example, results on the association between diabetes and CLD in the whole regional mortality archive were compared with those of a study on mortality from CLD in a cohort of diabetic subjects [5].

## Methods

### The death certificate and the selection of the UCOD

The certifying physician is required to report those conditions involved in the causal chain of events leading to death in Part I of the death certificate, and other significant conditions contributing to death in Part II. A proper certification in Part I (constituted by three or four lines, according to country and time period) would show a causal sequence from the immediate cause of death, to intermediate cause(s), to a single underlying cause of death, defined as the disease or injury which initiated the train of morbid events leading directly to death [17]. However, due to different types of inconsistencies in the certification (multiple diseases reported in the line reserved to the underlying cause, incorrect causal sequences), standard mortality statistics are based on internationally adopted algorithms which identify the UCOD from all the conditions reported in the certificate. The UCOD generally corresponds to the underlying cause stated by the certifier, but could also be another disease reported in Part I or Part II, or a derived condition [13].

The above algorithms are applied in an increasing number of countries by means of the Automated Classification of Medical Entities (ACME), which is a computer program developed by the National Center for Health Statistics to standardize assignment of the underlying cause. ACME has become an international standard, and many countries use the ACME decision tables as the basis for their own systems [20].

Specific guidelines on death certification recommending placement of diabetes in Part I or II of the certificates are lacking. According to existing coding rules, diabetes is selected as the UCOD when reported as a cause if ischemic heart diseases or cerebrovascular diseases in Part I of the certificate [3, 21]. When diabetes is selected as the UCOD, the fourth character of diagnosis codes should detail associated complications, such as cardiovascular diseases and renal failure; however, owing also to ambiguity of coding rules, most certificates are coded to “diabetes without complications” [3].

### The setting of the study

The Veneto region (northeastern Italy), has about 4.9 million inhabitants. Life expectancy is slightly higher than national figures, about 80 and 85 years in males and females, respectively. The main causes of mortality are represented by circulatory diseases and cancers [5]. The prevalence of self-reported diabetes is lower than overall Italian figures, but is rapidly increasing over time and is now approaching 5 % [22].

A copy of death certificates of each resident in the Veneto region is routinely transmitted to the Regional Epidemiology Department for coding of the causes of death according to the International Classification of Diseases, 10th Edition (ICD-10). Since 2008 the regional mortality database includes not only the UCOD, but all the diseases mentioned in the certificate, and the selection of the UCOD is performed using the ACME software.

### Analysis of diabetes-related mortality

ICD-10 codes for diabetes (E10-E14) were searched for in any position of the death certificates of the whole mortality archive through the period 2008 to 2010 in order to retrieve all diabetes-related deaths. According to the Part and line where diabetes was placed by the certifier on the death certificate, diabetes-related deaths were classified according to the following mutually exclusive categories: diabetes reported in the line reserved for the underlying cause, alone or in combination with other diseases; diabetes not chosen by the certifier as the underlying cause, but mentioned in another line of Part I; and diabetes not mentioned in part I, but only in Part II of the certificate. In each case, the proportion of deaths with diabetes selected as the UCOD by the ACME software was determined.

Age- and gender-specific mortality rates and proportional mortality were computed both for diabetes selected as the UCOD by ACME, and for deaths with any mention of the disease in the certificate. Population figures from the National Institute for Statistics [23] were the denominator of mortality rates. The ratio of mortality rates with diabetes mentioned and with diabetes selected (MCOD/UCOD) was computed.

### Mention of diabetes in death certificates of known diabetic subjects

Prevalence and determinants of mentioning diabetes were analyzed in a subset of death certificates of known diabetic subjects. Upon certification from a specialist, in Italy subjects with diabetes receive disease-specific care without any contribution to the costs: these patients are listed in a regional archive of exemptions from medical charges. This archive is estimated to include about 80 % of subjects identified as diabetic by multiple data sources, and can be used for the identification of a large cohort of diabetic patients; details have been previously published [5]. Briefly, records from 173,260 patients exempt from medical charges due to diabetes and alive on December 31, 2007 were linked to the mortality archive until December 31, 2010. The record-linkage was performed on previously anonymized records, without any possibility of identification of individuals.

The outcome dichotomous variable was mention of diabetes in the death certificate. Since it represented a common outcome in the study subjects, to estimate relative risks (RR) with 95 % Confidence Intervals (CI), instead of standard logistic regression, Poisson regression with a robust error variance was adopted both at univariate and multivariate analysis [24]. The explanatory variables retrieved from mortality records were gender, age at death, place of death, and mention on the certificate of selected diseases: hypertensive diseases (ICD-10 codes I10-I15), ischemic heart diseases (I20-I25), cerebrovascular diseases (I60-I69), neoplasms (C00-D48), renal failure (N17-N19), diseases of the respiratory system (J00-J99), and diseases of the digestive system (K00-K93). Furthermore, records of exemption from medical charges included the date of diabetes registration in the archive. This date could follow the diagnosis by several years, but allowed for the identification of a sub-cohort of patients (those registered before 2001) who definitely had a duration of diabetes of at least seven years prior to death. All the above study variables were included in the multivariate analysis.

### Association between diabetes and CLD in the death certificate

In the whole regional mortality archive, the association between diabetes and CLD was investigated as an example of the analysis of associations between chronic diseases mentioned in the certificate. Analyses were carried out on subjects aged 30–89 years, and CLD-related deaths were identified by codes K70, K73, K74 in any position of the death certificate. CLDs were further classified as related to viral hepatitis infection (codes B15-B19), to alcohol (codes for alcoholic liver diseases and for mental and behavioral disorders due to use of alcohol, K70 and F10), or with non-viral non-alcohol related etiology (NVNA-CLD, in the absence of the above codes).

To test the association between diabetes and CLD, any mention in the death certificate of CLD was the outcome variable, and any mention of diabetes was considered the exposure variable in a case–control analytic approach. To investigate if such association could reflect a real causal relationship at the population level, and to limit known bias in MCOD analyses [19], three different conditional logistic regression models were applied with CLD-related deaths as cases. We chose conditional logistic regression because we were not interested in model estimates for variables other than mention of diabetes in the death certificate, while taking into account stratification variables. The simplest regression (Model 1) was stratified by gender and age, with all non-CLD-related deaths as controls. In Model 2, controls were selected from conditions thought to be less strongly associated with diabetes than other disease categories: analysis was still stratified by gender and age, but controls were restricted to decedents from diseases of the respiratory system. Lastly, in order to take into account common opinions among physicians filling out the death certificate, Model 3 was stratified by the main variables demonstrated to be associated to the mention of diabetes in known diabetic subjects: age, gender, place of death, mention of selected circulatory diseases, and mention of neoplasms in the certificate. Controls were all non-CLD-related deaths. The analyses were repeated for all CLD, and for viral, alcoholic, and NVNA-CLD.

Results were compared with published findings from the above cohort of diabetic patients, where the same age interval was selected, the same disease definitions were adopted, and a higher mortality from CLD was found with respect to the general population. Through indirect standardization, standardized mortality ratios (SMR) with regional mortality rates as reference were significantly increased in diabetic patients and varied by etiology of liver disease [5].

## Results

### Analysis of diabetes-related mortality in the whole regional archive

Out of 132,511 deaths in the regional population in 2008 to 2010, diabetes was mentioned in 16,279 decedents (12.3 %); among the latter, diabetes was selected as the UCOD in 3,873 (2.9 % of overall deaths). The MCOD/UCOD ratio was 4.2. Table 1 shows that in approximately two-thirds of certificates, diabetes was mentioned only in Part II. In these cases, its selection as the UCOD was highly improbable, although it was still possible by the application of selection rules (specifically, Rule 3) [14]. When diabetes was reported in Part I on the underlying cause line, a large proportion of certificates (37 %) also mentioned other diseases on the same line. However, diabetes was selected as the UCOD in more than 90 % of certificates if reported alone or as the first entered condition on the line corresponding to the underlying cause. Moreover, diabetes was often selected as the UCOD when mentioned in other lines of Part I. Among 1,845 death certificates where diabetes was in other lines of Part I, the most frequent diseases reported as the first entered condition on the underlying cause line were: hypertensive (15 %), ischemic cardiac (19 %), cerebrovascular (9 %), and other circulatory diseases (13 %), and neoplasms (14 %, data not shown). In these cases, diabetes was selected as the UCOD in 68 % of certificates with a circulatory disease and in 31 % of certificates with a neoplasm on the underlying cause line.

**Table 1:** Selection of diabetes as the underlying cause of death, by part of the death certificate where the disease was mentioned: Veneto region (Italy), 2008-2010

	Death certificates with mention of diabetes	Death certificates with diabetes selected as the UCOD^a^	% of selection as the UCOD^a^
Part I	5,108	3,570	70
Part I, underlying cause line	3,263	2,468	76
Alone on the underlying cause line	2,040	1,890	93
Not alone, first entered on the underlying cause line	546	475	87
Not alone, other than first on the underlying cause line	677	103	15
Part I, other lines	1,845	1,102	60
Part II	11,171	303	3
TOTAL	16,279	3,873	24

^a^UCOD: underlying cause of death

**Fig. 1:**
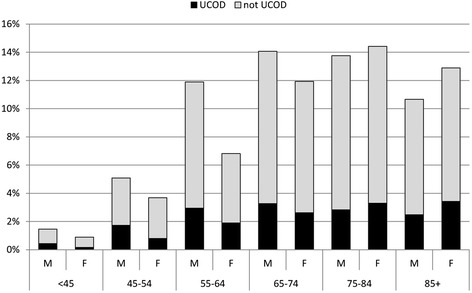
Proportional mortality by gender and age class from diabetes selected as the underlying cause of death (UCOD), or mentioned in any position of the death certificate: Veneto region, 2008–2010

Figure 1 shows proportional mortality and Table 2 reports mortality rates by gender and age class. It can be seen that both proportional mortality and mortality rates from diabetes were higher among males in younger age classes, but thereafter the increase with age was steeper among females. Such a tendency was found both in UCOD and MCOD analysis. The MCOD/UCOD ratio peaked in the 65–84 years age class, and was slightly higher in males (4.4) than in females (4.0).

**Table 2:** Mortality rates from diabetes in the Veneto region (Italy), 2008–2010: diabetes selected as the underlying cause of death, or as any mention in the death certificate (multiple causes of death)

	Underlying cause of death		Multiple causes of death		Multiple/underlying cause ratio
	n	Rate × 10^5^	n	Rate × 10^5^	
Males					
<45	10	0.3	34	0.9	3.4
45–54	45	4.3	132	12.5	2.9
55–64	182	21.0	736	84.9	4.0
65–74	428	61.2	1840	263.0	4.3
75–84	642	163.6	3121	795.2	4.9
85+	421	433.9	1812	1867.6	4.3
Total	1728	24.5	7675	108.7	4.4
Females					
<45	6	0.2	11	0.3	1.8
45–54	12	1.2	56	5.4	4.7
55–64	61	6.8	219	24.5	3.6
65–74	188	23.6	854	107.0	4.5
75–84	657	106.2	2869	463.6	4.4
85+	1221	459.4	4595	1729.0	3.8
Total	2145	29.0	8604	116.4	4.0
Males + females					
<45	16	0.2	45	0.6	2.8
45–54	57	2.7	188	9.0	3.3
55–64	243	13.8	955	54.2	3.9
65–74	616	41.1	2694	179.8	4.4
75–84	1299	128.5	5990	592.3	4.6
85+	1642	452.6	6407	1766.1	3.9
Total	3873	26.8	16279	112.6	4.2

### Mention of diabetes in a sub-set of known diabetic subjects

In the cohort of diabetic subjects, 19,605 deaths were identified. Diabetes was reported in any position in 52.1 % of death certificates, and selected as the UCOD in 13.4 %. The other main UCODs were circulatory diseases (36.2 % of deaths in the cohort) and neoplasms (28.3 %). Table 3 shows that diabetes was unlikely to be reported when the UCOD was a neoplasm, irrespective of the site of cancer. The mention of diabetes was higher with specific circulatory diseases selected as the UCOD (hypertensive, ischemic heart, or cerebrovascular diseases), even though in a substantial proportion of such deaths diabetes was not reported in any part of the certificate. At univariate analysis, the mention of diabetes increased with age, among females, in decedents at home, and in the sub-cohort with long disease duration; furthermore it varied by reported co-existing diseases (Table 4). At multivariate analysis, recording of diabetes was not associated with age, whereas this it was strongly increased when renal failure, hypertensive, ischemic heart, and cerebrovascular diseases were reported, and decreased in the presence of a neoplasm. Place of death, duration of disease, and, to a lesser extent, gender were still significantly associated with the mention of diabetes.

**Table 3:** Underlying cause of death in 19,605 known diabetic subjects, and proportion of death certificates with mention of diabetes. Veneto region (Italy), 2008-2010

Underlying cause of death (ICD-10 codes)	Number of deaths	Mention of diabetes (%)
Diabetes (E10-E14)	2,623	100
All circulatory diseases (I00-I99)	7,096	52
Hypertensive diseases (I10-I15)	773	66
Ischemic heart diseases (I20-I25)	3,090	55
Cerebrovascular diseases (I60-I69)	1,540	51
All other circulatory diseases	1,693	42
All neoplasms (C00-D48)	5,556	34
Colon, rectum and anus (C18-C21)	549	33
Liver and intrahepatic bile ducts (C22)	641	35
Pancreas (C25)	600	34
Trachea, bronchus and lung (C33-C34)	1,034	33
All other neoplasms	2,732	34
Respiratory diseases (J00-J99)	1,008	48
Digestive diseases (K00-K93)	878	45
All other causes of death	2,444	45

**Table 4:** Factors associated with mention of diabetes in death certificates of 19,605 known diabetic subjects. Veneto region (Italy), 2008-2010

	N	Diabetes mentioned (%)	RR (CI) unadjusted^b^	RR (CI) adjusted^c^
Age				
<45	49	43	0.76 (0.55 – 1.05)	0.96 (0.72 – 1.29)
45–54	251	43	0.75 ( 0.65 – 0.87)	0.95 (0.84 – 1.08)
55–64	1,395	46	0.82 (0.77 – 0.87)	1.00 (0.95 – 1.06)
65–74	4,008	47	0.83 (0.80 – 0.87)	0.97 (0.94 – 1.01)
75–84	7,568	52	0.92 (0.90 – 0.95)	1.00 (0.97 – 1.03)
85+	6,334	57	1.00	1.00
Gender				
Females	9,183	55	1.11 (1.08 – 1.14)	1.06 (1.03 – 1.09)
Males	10,422	50	1.00	1.00
Duration of disease				
>7 years	10,660	57	1.24 (1.21 – 1.27)	1.17 (1.14 – 1.20)
<7 years	8,945	46	1.00	1.00
Place of death				
Home	4,327	68	1.44 (1.40 – 1.48)	1.40 (1.37 – 1.44)
Other	15,278	47	1.00	1.00
Other diseases mentioned in the certificate				
Specific circulatory mentioned^a^	10,488	67	1.93 (1.87 – 1.99)	1.76 (1.70 – 1.82)
Not mentioned	9,177	35	1.00	1.00
Renal failure mentioned	3,661	63	1.27 (1.23 – 1.30)	1.26 (1.23 – 1.30)
Not mentioned	15,944	50	1.00	1.00
Tumors mentioned	6,658	39	0.67 (0.65 – 0.69)	0.85 (0.83 – 0.88)
Not mentioned	12,947	59	1.00	1.00
Respiratory diseases mentioned	6,356	53	1.02 (0.99 – 1.05)	1.06 (1.03 – 1.09)
Not mentioned	13,249	52	1.00	1.00
Digestive diseases mentioned	3,043	47	0.90 (0.86 – 0.93)	1.10 (1.06 – 1.14)
Not mentioned	16,562	53	1.00	1.00

^a^Hypertensive diseases, ischemic heart diseases, cerebrovascular diseases

^b^Risk Ratios with 95 % confidence intervals estimated by Poisson regression with a robust error variance

^c^Risk Ratios with 95 % confidence intervals adjusted for all the other variables reported in the Table, estimated by Poisson regression with a robust error variance

### Association between diabetes and CLD by different analytic approaches

Table 5 shows the association between diabetes and CLD in the whole regional mortality archive. In all analyses, if there was mention of CLD in the death certificate, the likelihood of diabetes being mentioned increased, supporting a causal relationship between the diseases. However, the strength of the association and differences by etiology of CLD varied according to the type of analysis performed. With respect to Model 1 (analysis stratified only by age and gender), all risk estimates were higher in Model 2 (controls restricted to respiratory deaths) and in Model 3 (analysis stratified by strong determinants of diabetes reporting in the certificate). Furthermore, in Model 2 and in Model 3 NVNA-related CLD displayed a higher risk with respect to CLD with viral or alcoholic etiology.

**Table 5:** Multiple causes of death analyses: Odds Ratio for mention of diabetes (vs. no mention) in certificates reporting chronic liver diseases, estimated by three different models of conditional logistic regression. Veneto region (Italy), 2008-2010

	Model 1	Model 2	Model 3
	OR (CI)^b^	OR (CI)^b^	OR (CI)^b^
All CLD^a^(n = 4,563)	1.59 (1.47 – 1.73)	1.69 (1.49 – 1.91)	1.91 (1.76 – 2.08)
Virus-related CLD^a^ (n = 1,103)	1.40 (1.19 – 1.65)	1.49 (1.23 – 1.81)	1.82 (1.53 – 2.15)
Alcohol-related CLD^a^ (n = 1,206)	1.68 (1.44 – 1.96)	1.62 (1.32 – 2.00)	1.79 (1.52 – 2.10)
NVNA-related CLD^a^ (n = 2,350)	1.67 (1.50 – 1.86)	1.82 (1.57 – 2.11)	2.06 (1.84 – 2.30)

^a^CLD = chronic liver diseases; 96 CLD were classified as related to both alcohol and viral hepatitis

^b^Odds ratio with 95 % Confidence Interval

Model 1: stratified by gender and age; controls = all non-CLD related deaths

Model 2: stratified by gender and age; controls = deaths with respiratory diseases as the underlying cause

Model 3: stratified by gender, age, place of death, mention of specific circulatory diseases, and mention of cancer; controls = all non-CLD related deaths

## Discussion

The study provides a comprehensive picture of the use of MCOD in investigating the mortality burden related to a chronic disease. The first issue regards diabetes reporting in death certificates of decedents affected by the disease. Physicians may not record diabetes on the death certificate because they could be unaware of the disease in the patient, may not consider that diabetes contributed to the patient’s death, or may not have listed diabetes because of space constraints [7]. Physicians completing death certificates mention only conditions considered to be instrumental in causing death, not all prevalent diseases at death [25]. According to a review, the median proportion of diabetes reporting in any position of the death certificate among decedents with known diabetes was only 43 % [26]. The recording of diabetes in US death certificates did not increase from 1986 to 1993, being less than 40 % among decedents with a history of diabetes [6]. In the TRIAD study, 39 % of diabetic subjects had the disease recorded; diabetes was less frequently reported for all causes of death other than cardiac diseases, especially cancer [7]. In the US, the mention of diabetes was higher if the certifying physician was the primary care physician [8], or if the place of death was the patient’s home [9]. In studies from the UK, diabetes was mentioned in 42-43 % of death certificates of known diabetics, being associated with an increased duration of diabetes and insulin treatment, increasing age, female gender, low social class, and a cardiovascular underlying cause of death [10, 11]. The present findings show a higher proportion of diabetes reporting (52 %) with respect to studies from the US or the UK. A problem of over-designation of diabetes as a cause of death exists if the certifying physician lists all diseases affecting the decedent, without considering their real role in causing the death. On the other hand, the present data show that diabetes was not mentioned in a substantial proportion of certificates where cardiovascular disorders such as ischemic heart diseases were selected as the UCOD. Furthermore, the study confirms that many variables are associated with the mention of diabetes: patient’s gender, long duration of disease, circumstances of death (for subjects dying at home certificates are generally filled by the family doctor or by a community health physician), and co-existing diseases leading to death. Unfortunately, we did not have data to test the role of diabetes treatment on the mention of the disease (no treatment, only oral anti-diabetic drugs, insulin). Moreover, we had no measure of the specificity of diabetes reporting; according to the few studies available in the literature, specificity was 98 % in the Rancho Bernardo study [9], and the positive predictive value was 99 % in a sample of death certificates in France [27].

The second major issue is the quality of cause-of-death statements in certificates with mention of diabetes. It is often difficult for the certifying physicians to decide whether to report diabetes in Part I of the certificate, which indicates that diabetes directly caused death, or in Part II, which suggests that diabetes contributed to death but was not part of the sequence of events directly leading to death [20]. As an example, Taiwanese physicians were much more likely to report diabetes in Part I (70 %) than their counterparts in Sweden (21 %) and in the US (36 %) [12]. When diabetes is recorded in Part I, there are two major types of improperly filled cause-of-death statements: reporting more diagnoses per line, and incorrect causal sequence among the reported diagnoses, usually resulting in an UCOD selected by the ACME software different from the underlying cause chose by the certifying physician. In previous studies, about three-quarters of the incorrect causal sequences involved incorrectly reporting other conditions as the cause of diabetes, mainly hypertension and acute myocardial infarction. A less frequent anomaly was diabetes incorrectly reported as the cause of other diseases [15, 28]. In our database, diabetes was most frequently mentioned in Part II of the certificate. When diabetes was reported on the line reserved for the underlying cause in Part I as the only or the first reported condition, it was selected as the UCOD in most cases. Based on ACME decision tables, many diseases can be the consequence of diabetes, and rejected causal sequences starting with diabetes as the underlying cause were rare. The other types of error were much more frequent: reporting multiple diseases per line, and diabetes mentioned in other lines of Part I as due to other diseases, mainly selected circulatory diseases. In these latter cases, which can be regarded as a major flaw of death certification practices, diabetes was frequently selected as the UCOD. A main issue is the lack of training of the certifying physician. The development of electronic certification has been proposed to facilitate the process with online explanations, and to limit errors when completing the death certificate [29]. However, due to the ageing population (the median age at death in the Veneto region was 78 years in males and 85 in females) and the associated increase in the incidence of multiple comorbid conditions, there may be no simple etiologic chain leading to the identification of a single underlying cause; instead, death often results from a complex interaction between multiple factors [17, 29].

Within this context, official mortality data for chronic diseases should be provided based both on the UCOD and on MCOD. Age-specific mortality rates based on the UCOD were similar to those reported in the literature, but among older age classes, rates based on MCOD tended to be higher than previous reports. The ratio between diabetes reported as MCOD/UCOD displays heterogeneity by country: 4.2 in our study, 2.6 in France, 4.2 in UK, 4.5 in Sweden, and 3.1 in the US [30]. Overall, diabetes was mentioned as MCOD in a larger proportion of overall deaths (12.3 %) than reported in other countries: 5.3 % in France in 2002 [30], 5.1 % in England in 1995–2010 [31], 10.6 % in Canada in 2004–2008 [32], and 9 %, 10 %, 9 % in 2000–2001 in Sweden, Taiwan, and the US, respectively [12]. This finding might be due to more recent data and an older population analyzed in the present study, and to a higher propensity to report diabetes in death certificates of elderly subjects.

The last issue is the association between different diseases mentioned in the death certificate. Different measures of association have been proposed in the literature [33]. The simplest analysis would involve examining the frequency with which two conditions are reported together; estimation of Odds Ratios (OR) stratified by confounding factors such as age is usually necessary [19]. We compared results on the association between diabetes and CLD in the regional mortality archive (Table 5) with those from the cohort of subjects exempt from medical charge [5]. In this cohort, mortality from all CLD was higher than in the general population in both analyses restricted to the UCOD (SMR = 2.55), and in MCOD analysis (SMR = 2.55). Results remained unchanged if analyses were restricted to diabetics with long disease duration, confirming a role of diabetes in increasing the mortality from CLD. In MCOD analyses, mortality was higher for NVNA-CLD (SMR = 2.86) than for alcohol- (SMR = 2.25) or virus-related CLD (SMR = 2.17) [5], a finding similar to Model 2 and 3 of the present results.

Redelings and colleagues have already reviewed possible bias affecting ORs estimated from mortality data [19]. Any exposure that increases the likelihood that someone will die also increases the likelihood of inclusion in the study, generating a type of selection bias termed Berkson’s bias. Furthermore, chronic conditions are rarely reported in death certificates without multiple associated diseases, increasing the likelihood of spurious associations between them. Selection bias can be limited by choosing controls who died from diseases unrelated to the exposure [19]. However, since diabetes increases the mortality risk from many causes, our first choice was not to restrict the selection of controls (Model 1); an alternative strategy was to select deaths from respiratory diseases (Model 2). Another strong bias possibly acting in our database was reporting bias due to common opinions among physicians completing death certificates, based on the biological plausibility of a connection between diseases [19]. In our study, the mention of diabetes was increased in the presence of renal failure and selected circulatory diseases, and decreased by the presence of cancer (including liver cancer, often arising from CLD). This latter observation could be extended to many chronic diseases, since the overall prevalence of reported comorbid conditions is usually lower in deaths with an underlying neoplastic cause [34, 35]. A further analytic strategy was therefore to stratify by major factors associated with diabetes reporting (Model 3); however, this could have led to overmatching bias [19]. Furthermore, we had no data on completeness of the mention of CLD in mortality records. In view of all the above, caution is needed in examining associations observed between diseases in MCOD data, and multiple analytic strategies, such those adopted in the present study, should be explored to confirm results. The association between diseases reported in the death certificate depends on a complex interplay between different factors: the real etiological relationship between the diseases, current medical knowledge and beliefs, and certification practices. Such analyses could be useful to generate etiologic hypotheses to be assessed by other study designs. In our experience, preliminary findings from the MCOD archive showing the association between diabetes and CLD led us to perform the cohort mortality study.

The present study has both strengths and limitations in exploring the potential of MCOD analyses. Among the strengths, the study was carried out on mortality data coded according to ICD-10, with selection of the UCOD carried out by the ACME software. To our knowledge, this is the largest study investigating rate and factors associated to diabetes reporting in death certificates. Moreover, findings on the association between diseases mentioned in the certificate were compared with results of a cohort study. Among the limitations, we did not have a direct measure of the negative predictive value and the specificity of reporting diabetes in death certificates. Lastly, as in other countries [1], over half of all diabetes deaths reported the code E149 (unspecified diabetes mellitus without mention of complications); therefore, examination of the breakdown of specific diabetic codes was not performed. It is worth noting that the selected UCOD is also often “diabetes without complication” when diseases such as renal failure are mentioned in the certificate. In these circumstances analytic strategies based on MCOD are essential to capture the real burden of mortality from diabetic renal disease [36].

As a final remark, a paper published more than fifty years ago stated that although mortality statistics were not intended to give a comprehensive picture of disease prevalence among decedents, data based on MCOD were more informative than usual UCOD tabulations, especially for chronic conditions such as diabetes. Furthermore, some diseases, including liver cirrhosis, were found to be reported in association with diabetes more frequently than in overall deaths [37]. In spite of these old observations, such analyses are applied in few countries, and the scientific literature on the potential and intricacies of MCOD remains limited.

## Conclusion

MCOD conveys much more information than usual mortality statistics based only on the UCOD. MCOD analyses not only allow more complete assessment of the burden of mortality related to chronic diseases, but are also useful to monitor patterns and inconsistencies in cause of death certification. Furthermore, associations between diseases reported in death certificates can be explored with the aim to generate etiologic hypotheses to be assessed by other study designs.
